# A disease spectrum for *ITPA* variation: advances in biochemical and clinical research

**DOI:** 10.1186/s12929-016-0291-y

**Published:** 2016-10-22

**Authors:** Nicholas E. Burgis

**Affiliations:** Department of Chemistry and Biochemistry, Eastern Washington University, 226 Science Building, Cheney, WA 99004 USA

**Keywords:** ITPA, ITPase, Inosine, ITP, Ribavirin, Azathioprine, Thiopurine, Hepatitis C, Infantile encephalopathy, Tuberculosis

## Abstract

Human ITPase (encoded by the *ITPA* gene) is a protective enzyme which acts to exclude noncanonical (deoxy)nucleoside triphosphates ((d)NTPs) such as (deoxy)inosine 5′-triphosphate ((d)ITP), from (d)NTP pools. Until the last few years, the importance of ITPase in human health and disease has been enigmatic. In 2009, an article was published demonstrating that ITPase deficiency in mice is lethal. All homozygous null offspring died before weaning as a result of cardiomyopathy due to a defect in the maintenance of quality ATP pools. More recently, a whole exome sequencing project revealed that very rare, severe human *ITPA* mutation results in early infantile encephalopathy and death. It has been estimated that nearly one third of the human population has an *ITPA* status which is associated with decreased ITPase activity. *ITPA* status has been linked to altered outcomes for patients undergoing thiopurine or ribavirin therapy. Thiopurine therapy can be toxic for patients with *ITPA* polymorphism, however, *ITPA* polymorphism is associated with improved outcomes for patients undergoing ribavirin treatment. *ITPA* polymorphism has also been linked to early-onset tuberculosis susceptibility. These data suggest a spectrum of *ITPA*-related disease exists in human populations. Potentially, *ITPA* status may affect a large number of patient outcomes, suggesting that modulation of ITPase activity is an important emerging avenue for reducing the number of negative outcomes for *ITPA*-related disease. Recent biochemical studies have aimed to provide rationale for clinical observations, better understand substrate selectivity and provide a platform for modulation of ITPase activity.

## Background

**Fig. 1:**
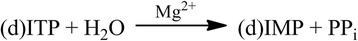
ITPase reaction scheme

Inosine triphosphate pyrophosphatase (ITPase) has a critical role in sanitizing nucleotide pools by removing (deoxy)nucleoside triphosphates ((d)NTPs) containing noncanonical purines [1–7]. ITPase is a pyrophosphohydrolase and converts noncanonical purine (d)NTPs, such as (deoxy)inosine 5′-triphosphate ((d)ITP), into their corresponding nucleoside monophosphate ((d)NMP); releasing pyrophosphate (PP_i_) [8] (Fig. 1). Noncanonical purine (d)NTPs spontaneously arise in cells. In the purine biosynthesis pathway, phosphorylation of inosine 5′-monophosphate (IMP), a precursor to adenosine 5′-monophosphate (AMP) and guanosine 5′-monophosphate (GMP), can produce both ITP and dITP [9, 10]. Additionally, oxidative deamination of adenine containing nucleosides/nucleotides or deamination of guanine containing nucleosides/nucleotides results in the formation of inosine containing nucleosides/nucleotides [11]. In general, evidence supports that ITPase, and its orthologs, prevent the accumulation of noncanonical purine (d)NTPs and are conserved throughout the three domains of life and are even found in viruses [2–4, 12–16]. Research with bacterial, yeast and murine systems has demonstrated that ITPase deficiency and/or polymorphism can result in sensitivity to noncanonical purines, increased mutation rates, delayed cell cycle progression, DNA damage and chromosomal abnormalities such as double-strand breaks [17–21].

ITPase is expressed in all major adult human tissues [5]. Results from Northern blot and cDNA microarray data indicate the highest expression is in the heart, liver, thyroid and thymus [5]. Likewise, mouse *Itpa* is expressed in all major tissues, however the highest expression levels were found to be in the testis, brain and thymus [22]. Mouse *Itpa* expression is driven by a TATA-less promoter which has characteristics that suggest it is a ubiquitously expressed housekeeping gene whose expression can be amplified under certain conditions [22].

**Fig. 2:**
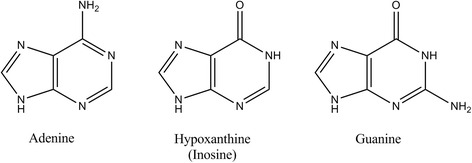
Structure of purine bases. Hypoxanthine is the nucleobase of inosine

Studies in a mouse model suggest that the role of ITPase is highly pronounced in cardiac tissues [6]. Cardiomyopathy in ITPase knock-out mice appears to result from the accumulation of ITP in the nucleotide pools and subsequent utilization of the contaminated pool. Analysis of the nucleotide content of total heart RNA revealed high levels of IMP which were roughly equivalent to 1 % of the AMP level whereas IMP was undetectable in wild-type total RNA samples [6]. Similarly, increased levels of dIMP were observed in DNA harvested from embryos deficient in ITPase [18]. The structural similarities between adenine, guanine and hypoxanthine (hypoxanthine is the nucleobase of ITP) (see Fig. 2), make it plausible that ITP can act as an improper substrate and replace canonical nucleotides in some processes. One study has shown that actomyosin binding of ITP in place of ATP reduces the shortening velocity and rate of force recovery and results in disordered striation during activation [23]. Therefore, an increase in ITP levels could affect actomyosin function, leading to disorganization of sarcomere structure in the developing heart. It is likely that the accumulation of (d)IMP in heart nucleic acids leads to the production of DNA lesions, defective proteins, nonfunctional RNAs and reduced cell proliferation [6, 18]. Because organ development among mammals is very similar [24], these observations should be highly relevant to humans. The critical role for ITPase in the heart is underscored by the observation that the heart is among the four tissues with the highest *ITPA* expression in humans [5].

For any cell in any organism one can imagine that genetic polymorphism could modulate purine metabolism and alter the amount of: hypoxanthine entering the purine salvage pathway, IMP produced from de novo purine biosynthesis, or phosphorylation/reduction of IMP to (d)ITP [25]. In any of these cases, proper function of ITPase would be vital to insure that inosine is excluded from the nucleic acids and nucleotide pools. Concurrent polymorphism in ITPase may lead to pleiotropic outcomes such as DNA damage, interference with gene expression and RNA function, and altered activity for nucleotide binding proteins [6, 17]. Additionally, it is possible that conditions which exacerbate nitrosative stress, such as inflammation, would result in increased deamination of adenine and guanine containing nucleosides and nucleotides; resulting in increased concentrations of (d)ITP [26]. Support for these ideas stem from data we published in 2012 showing that perturbation of purine metabolism in *Escherichia coli* leads to substantial incorporation of (deoxy)inosine in nucleic acids [17]. Furthermore, an association between *ITPA* polymorphism and mitochondrial dysfunction has been reported, suggesting nucleotide imbalances may alter mitochondrial function [27]. Altogether, ITPase is an essential protector of homeostasis in the cell, and most likely reduces the occurrence of genome instability, cancer, ageing, and disease in humans.

Below I will summarize several recent studies concerning ITPase biochemistry and clinical outcomes associated with *ITPA* polymorphism or mutation. The goal of this review is to: (1) strengthen the connection between basic science and clinical studies; (2) summarize the many disparate clinical outcomes that have recently been linked to *ITPA* polymorphism/mutation; (3) argue in favor of viewing *ITPA* variation as a disease spectrum; and (4) suggest rationale for modulation of ITPase activity in an effort to reduce the number of negative outcomes for *ITPA*-related disease.

### Biochemistry

ITPase is a pyrophosphohydrolase that excludes noncanonical purines from the (d)NTP pools by hydrolyzing the acidic anhydride bond between the alpha and beta phosphates of the incoming (d)NTP (Fig. 1) [8, 28, 29]. A key step in the enzyme mechanism appears to be substrate binding, and ITPase only has high affinity for NTPs containing noncanonical purines (ITP, dITP and xanthosine 5′-triphosphate) [2, 5, 8]. Tight binding of the noncanonical nucleobase in the nucleobase binding pocket of the enzyme allows proper alignment of distal catalytic residues to catalyze hydrolysis of the phosphoanhydride bonds, while interactions with NTPs containing canonical bases result in improper alignment of the proposed catalytic residues and the scissile phosphoanhydride bond, and a much lower rate of catalysis [2–4].

ITPase is an α/β homodimeric protein [4]. Each monomer consists of 194 amino acid residues and the protein forms a 45 kDa dimer [5]. The protein has a central elongated mixed β-sheet which forms a platform to support two globular lobes [4]. ITP binds in a cleft, between the lobes, adjacent to the dimer interface. The specificity pocket is buried deep within the dimerization lobe of each monomer while the catalytic site is toward the periphery, close to the dimer interface in the N-terminal lobe [4]. Catalysis requires Mg^2+^ [28, 30], and is thought to occur via acid-base chemistry [3, 4], although the specific mechanism has not been elucidated. Maximal activity has been observed with alkaline pH and in the presence of reducing agent such as dithiothreitol (DTT) [5, 28] (see reference [7] for a review of biochemical studies).

ITPase does not discriminate between the ribose and deoxyribose carbohydrate moiety, and hydrolyzes phosphoanhydride bonds in noncanonical NTPs and dNTPs with equal activity [2, 5]. One study has reported strong positive cooperativity for the *E. coli* ITPase ortholog, RdgB [13], however, cooperativity has not been reported elsewhere. Human ITPase is competitively inhibited by inosine 5′-diphosphate, but not NMPs or any nucleoside tested [8]. Interestingly, strong substrate inhibition has been observed in multiple studies using purified ITPase in the presence of elevated levels of ITP or dITP [2, 5, 28]. The enzyme has also been shown to be inhibited by Cd^2+^, Co^2+^ and Ca^2+^ ions [29].

**Fig. 3:**
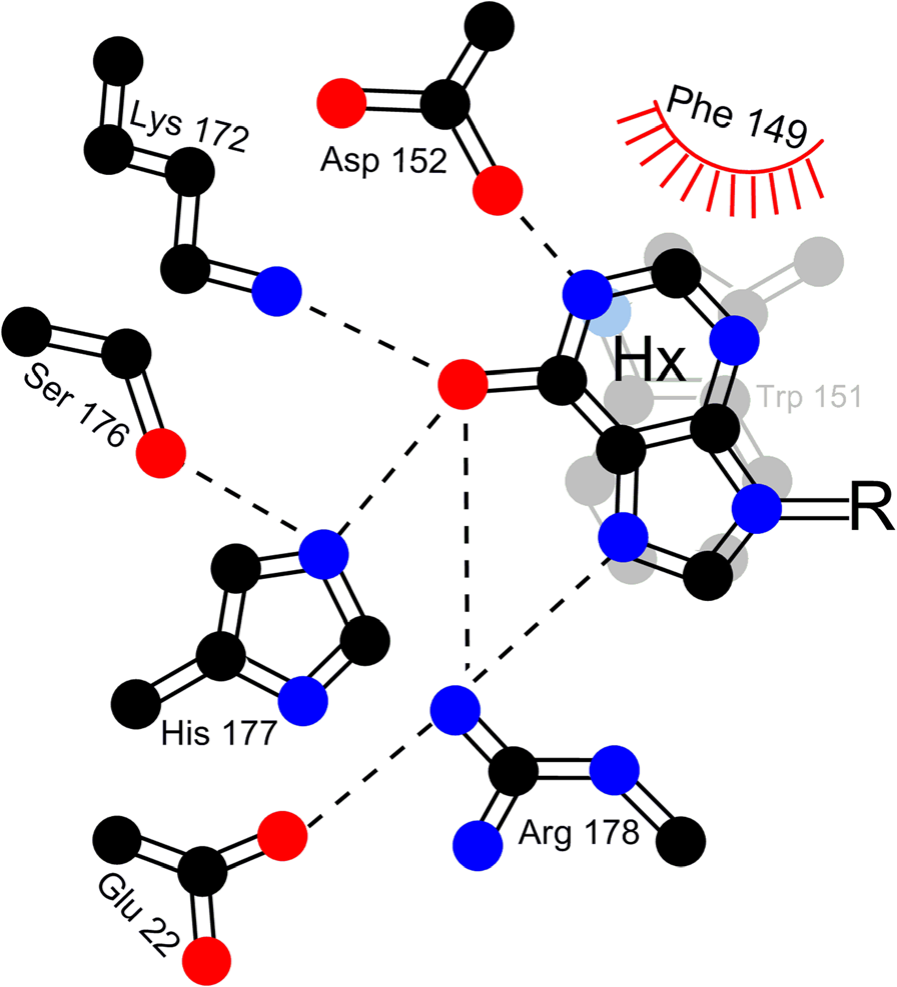
Model of ITPase substrate binding pocket. CPK coloring. Hx, hypoxanthine base of inosine; R, sugar (ribose) ring. *Dashed lines* indicate putative hydrogen bonds. Image reproduced from reference [31] with permission

ITPase has been studied for over 40 years [28], however, the mechanism employed by ITPase to discriminate between canonical and noncanonical (d)NTPs is not fully understood. X-ray crystal structures of the protein-substrate complex have been solved [3, 4], and several evolutionarily conserved residues have been shown to govern substrate selectivity [31], but rigorous biochemical validation of the predictions have not been performed. The ITPase substrate specificity pocket is shown in Fig. 3.

An initial site-directed mutagenesis screen of the substrate specificity pocket supported several assertions based on the crystal structure [4, 31]. Experimental design of this screen allowed differences in specific activity to reflect differences in *K*_M_ and provide surrogate measurements of substrate binding [31]. Based on these measurements it seems that ITPase favors NTPs containing electronegative atoms at the 6-position, such as ITP and GTP over NTPs containing electropositive atoms such as the 6-position amine of ATP. Indeed, x-ray crystallography data suggest that three binding pocket residues have potential for making favorable hydrogen bonding interactions with the electronegative 6-postion carbonyl of ITP or GTP [4], however, biochemical data indicates Arg-178 as the most crucial residue for this discrimination [31]. X-ray data places Arg-178 within 3 Å (hydrogen-bonding distance) of both the 6-positon carbonyl oxygen and 7-position ring nitrogen atoms of the nucleobase in ITP. In this position Arg-178 would form favorable hydrogen bond interactions with ITP or GTP, but not ATP, which may account for its essential role in ITP detoxification and substrate selectivity [4]. Additionally, mutation of Arg-178 to alanine, or even the conservative arginine to lysine substitution (R178K), prohibits complementation of ITPase defects in *E. coli* in two in vivo tests. Hence, Arg-178 has been classified as essential for ITPase activity [31]. A substitution to cysteine at this position was identified in a patient suffering from infantile encephalopathy [32] and will be discussed below.

The ITPase nucleobase binding pocket adopts a topography that is exquisitely complementary to the incoming hypoxanthine base of ITP [3, 4]. While the mechanism for discrimination against adenine containing nucleotides lies primarily in hydrogen bonding capacity, discrimination against guanine containing NTPs appears to be governed by steric hindrance [4, 31]. Phe-149 is the major determinant for excluding GTP from the binding pocket. Mutation to alanine results in a roughly 6-fold increase in hydrolysis of GTP, and a lower level of complementation for ITPase defects in *E. coli*. Phe-149 is positioned in close proximity to the 2-positon ring carbon of the nucleobase. In GTP, this position is bonded to an exocyclic amine, which is absent in ITP. Thus Phe-149 actively excludes GTP from entering the binding site, and prevents proper alignment of the catalytic residues with the scissile phosphoanhydride bond [4, 31]. Overall, site-directed mutation experiments indicate that modifications which cause slight enlargements of the nucleobase binding site, reduce the level of discrimination afforded by the enzyme.

To date, ITPase biochemistry remains understudied; however, some advances have been made concerning substrate selectivity [31] and a clinically relevant variant [33–35]. Knowledge gaps exist in the biochemical understanding of ITPase and its specific role in purine metabolism [36]. For example, it is unknown if ITPase has protein-binding partners, if the enzyme is posttranslationally modified, or if it is allosterically regulated. Additionally, the role of substrate inhibition [2] in cellular function is vastly unknown and may serve to modulate the intracellular concentration of ITP, IMP or even inosine. Support for this idea lies in the discrepancy among data obtained from assays using either purified/recombinant ITPase or whole-cell extracts. For example, Bakker et al. found that ITPase is not subject to substrate inhibition when erythrocyte cell extracts are used as the protein source [37], whereas enzymology studies using purified/recombinant ITPase as the source of protein reported substrate inhibition [2, 5, 28]. Taken together, these results hint that the level of substrate inhibition for ITPase may be modulated in vivo. Overall, a better understanding of substrate inhibition may serve as a fruitful avenue for therapeutic modulation of ITPase activity (see below). Importantly, for one clinically significant variant, c.94C > A (p.Pro32Thr), a clear biochemical rationale for decreased enzyme activity is known (see below) [33–35].

### Clinically relevant *ITPA* variants/mutants

Over the last decade the pharmacogenetic significance of *ITPA* variation has been established. In humans, at least thirty polymorphisms have been identified in the *ITPA* gene [32, 38–44]. To date, seven of the variants have been shown to be clinically relevant (see Table 1) [32, 40–42, 44]. Multiple reports have demonstrated that *ITPA* status affects outcomes of hepatitis C treatment [45, 46] and thiopurine therapy [36, 47]. Additionally, *ITPA* mutation has been shown to cause early infantile encephalopathy [32] and variation has been linked to young-onset tuberculosis susceptibility [44]. Therefore, the spectrum of phenotypes related to *ITPA* status is full range and spans improved therapeutic outcomes to infantile death.

**Table 1:** Clinically relevant *ITPA* polymorphism

Location	SNP ID	variation	biological significance	Clinical significance	Reference
c.94C > A (p.Pro32Thr)	rs1127354	SNP	reduced expression, stability, catalysis	ADR	[40]
c.124 + 21A > C	rs7270101	SNP	poor splicing efficiency	ADR	[40]
c.264-607_295 + 1267del1906	NA	deletion	1,874 bp deletion, frameshift, nonfunctional protein	encephalopathy	[32]
c.359_366dupTCAGCACC (p.Gly123Serfs)	rs863225424	duplication	Frameshift, nonfunctional protein	encephalopathy	[32]
c.452G > A (p.Trp151Stop)	NA	nonsense	Nonsense RNA-mediated decay	encephalopathy	[32]
c.532C > T (p.Arg178Cys)	NA	SNP	Altered substrate specificity, poor solubility^a^	encephalopathy	[32], NEB^a^
g.19176G > A	rs13830	SNP	3′UTR variation; altered mRNA metabolism/translation	TB susceptibility	[44]

*ADR* adverse drug reaction, *NA* not available

^a^unpublished results

In 2004 it was estimated that about 5 % of the world population harbors the c.94C > A (p.Pro32Thr) ITPA variation with the highest penetrance, 14 to 19 %, in Asian populations [48]. Since then several more polymorphisms have been identified [32, 40, 44] (see Table 1) and recent reports suggest that nearly a third of the population may harbor *ITPA* polymorphism that is associated with decreased ITPase activity [49–51]. In fact, even heterozygous variation which results in overall ITPase activity that is 60 % of the homozygous wild-type level can be clinically significant [49, 51–54], however, pathological mutations are very rare [32].

Genotype status can result in a varying degree of reduction in ITPase activity. Most studies have focused on ITPase activity in erythrocytes for the more common polymorphisms, c.94C > A (p.Pro32Thr) and c.124 + 21A > C, and the results between studies are in good agreement [38–40]. For example, in Caucasian populations, the c.94C > A (p.Pro32Thr) polymorphism results in severe reduction of ITPase activity and the mean activity for heterozygous individuals is about 25 % of the wild-type levels while homozygous individuals retain less than 1 % of the wild-type levels [39]. The c.124 + 21A > C variant results in a more moderate reduction in ITPase activity, and heterozygotes retain about 60 % of the wild-type levels while homozygous individuals retain about 30 % of the wild-type levels. Consequently, c.94C > A (p.Pro32Thr) and c.124 + 21A > C compound heterozygosity results in severe reduction of ITPase activity and these individuals retain about 8 % of the wild-type levels [39]. It is important to underscore that these data are obtained from erythrocytes, and that the level of activity observed may be different for different cell types within the same individual.

#### ITPA variation/mutation: effects on protein structure and expression

Clinically relevant *ITPA* variants are listed in Table 1. Of the seven variants, four are single nucleotide substitutions, one a nonsense mutation, along with a duplication and a deletion. The later three instances are very rare and can be considered mutations as opposed to variants. These three mutants were identified in a cohort of homozygous patients suffering from infantile encephalopathy [32]. These mutants are predicted to have the greatest effect on protein structure/function by changing the length of the 194 residue protein and most likely result in a nonfunctional protein. The nonsense mutation (c.452G > A) introduces a premature stop codon at amino acid position 151, resulting in a C-terminal truncated protein, which would lack the highly conserved specificity pocket containing the SHR signature sequence at positions 176–178. For the duplication mutant (c.359_366dupTCAGCACC), an 8 bp duplication is introduced which results in a frameshift starting at amino acid positon 123. This mutation is predicted to produce a 225 amino acid polypeptide with altered amino acid sequence. Concerning the deletion mutation (c.264-607_295 + 1267del), a deletion of 1,874 base pairs was identified which completely spans exon 5 and is predicted to result in an out of frame transcript. ITPase activity in erythrocyte or fibroblast cells derived from patients homozygous for each of these three mutations was severely diminished, indicating significant loss of activity for the protein [32].

Of the four single nucleotide variants/mutants that are clinically relevant, two affect protein structure. Among these the c.94C > A (p.Pro32Thr) variant is the most studied. This common point mutation is thought to affect the protein in three different ways: decreased expression of the full length transcript, reduced catalytic activity and decreased stability [33–35, 55]. This variation has been shown to alter mRNA splicing events such that exon 2 and 3 are misspliced; an activity which presumably results in a nonfunctional protein. While this missplicing event happens in wild-type cells to some extent, the level of missplicing increases over three-fold for homozygous variants [55]. Additional studies have shown that substitution of proline with threonine at position 32 results in a protein with nearly two-fold less catalytic activity and reduced stability [33–35]. This position in the polypeptide chain is located in an exterior loop that is adjacent to the protein-dimer interface and close to residues that are thought to be involved in enzyme catalysis [3, 4]. Proline at this position is thought to have a role in restricting flexibility of the external polypeptide chain, which reduces ability of solvent to infiltrate the protein interior and helps maintain proper positioning of catalytic residues [33, 34]. Hence, this single nucleotide polymorphism (SNP) results in poor expression of an unstable, catalytically compromised protein.

The c.532C > T (p.Arg178Cys) mutant is very rare and has been recently identified in a patient suffering from infantile encephalopathy [32]. Arg-178 is a highly conserved position in the ITPase structure and is one of the residues that form the SHR signature sequence for ITPase. It is thought to have a major role in substrate selectivity as it is postulated to interact with the nucleobase of incoming (d)ITP at two positions [4] (see above). This particular variant has been predicted to result in a nonfunctional protein by multiple software programs [32]. Additionally, a site-directed mutagenesis study has identified this position as essential for ITPase activity both in vitro and in vivo [31].

The two additional single nucleotide variants do not affect protein structure. The common c.124 + 21A > C variant is an intronic substitution that prevents the canonical spliceosome reaction [55]. Substitution of adenine at this position is thought to destroy the preferred branch point in the splicing reaction, resulting in an alternative splicing event that is less efficient, therefore reducing expression of the *ITPA* gene [55]. The recently identified g.19176G > A variant is also thought to alter *ITPA* expression [44]. This particular variant is located in the 3′-untranslated region (3′-UTR) of the *ITPA* transcript. It has been hypothesized that this variant affects mRNA metabolism and/or translation resulting in a higher level of ITPase expression [44].

### Clinical effects of *ITPA* variation/mutation

#### Role of ITPase in drug metabolism

**Fig. 4:**
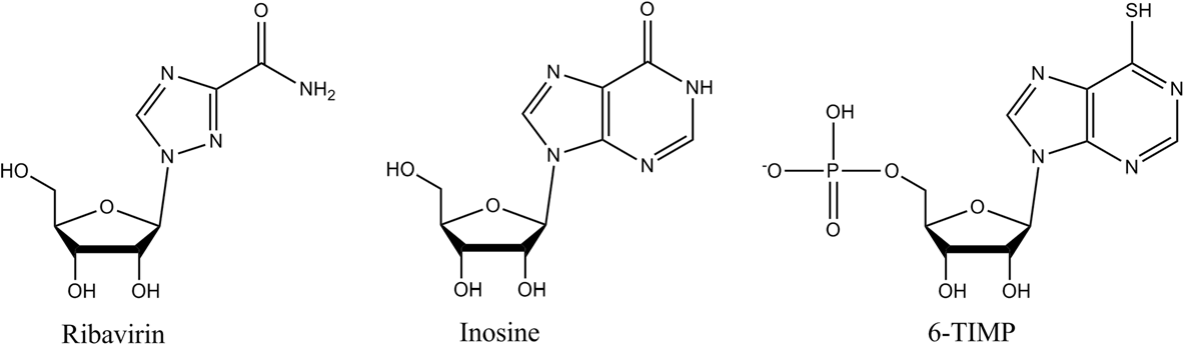
Comparison of relevant nucleoside/nucleotide structures. Ribavirin is bioactivated to the triphosphate form; inosine is an intermediate in purine metabolism; 6-TIMP (6-thio-inosine 5′-monophosphate) is an intermediate in thiopurine bioactivation

**Fig. 5:**
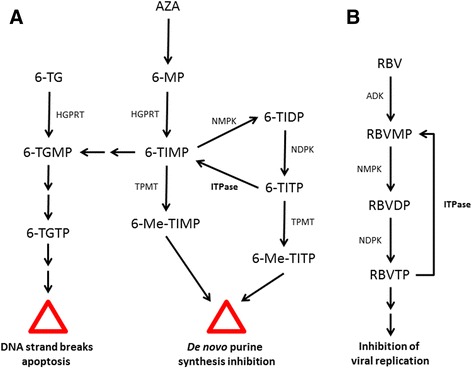
Pathways for drug bioactivation and drug degradation by ITPase. **a** Thiopurine metabolism. Based on [7, 36]. **b** Ribavirin metabolism. AZA: azathioprine; 6-TG, 6-thioguanine; 6-MP: 6-mercaptopurine; 6-TIMP, 6-TIDP, 6-TITP: 6-thio-inosine 5′-mono-, di- and tri-phosphate; 6-TGMP, 6-TGTP: 6-thio-guanosine 5′-mono- and tri-phosphate; 6-Me-TIMP, 6-Me-TITP: 6-methylthio-inosine 5′-mono- and tri-phosphate. RBV: ribavirin; RBVMP, RBVDP, RBVTP: ribavirin 5′-mono-, di- and tri-phosphate; HGPRT: hypoxanthine-guanine phosphoribosyltransferase; TPMT: thiopurine *S*-methyltransferase; NMPK, NDPK: nucleoside mono- and di-phosphate kinase; ADK: adenosine kinase

ITPase is thought to have a direct role in metabolism of thiopurine drugs [36]. Thiopurines such as azathioprine and 6-mercaptopurine are prodrugs and require enzymatic bioactivation to undergo ribosylation and phosphorylation to become the active nucleotide form. In the active form, thiopurines are antimetabolites which act by replacing guanosine containing nucleotides in physiological processes. Hence, thiopurines can disrupt multiple biologic pathways such as DNA and RNA metabolism, purine metabolism, and stimulate pro-apoptotic pathways (see references [56, 57] for review). One intermediate in this pathway, 6-thio-inosine 5′-triphosphate (6-TITP), has a structure nearly identical to ITP (see Fig. 4 for comparison of nucleobases). 6-TITP has been shown to be a substrate for ITPase [36]. This result suggests that the drug may enter a futile cycle of phosphorylation by kinase enzymes to the triphosphate form, and hydrolysis by ITPase back to the monophosphate form. Therefore, the end result is that the ITPase enzyme acts to reduce an intermediate in the pathway, and subsequently the amount of active form of the drug present in human cells (Fig. 5a).

Ribavirin is also activated by intracellular kinases to form a nucleoside triphosphate (Fig. 5b) [52, 58]. In this case, the ribonucleoside triphosphate becomes a substrate for the replicative RNA-dependent RNA polymerase and it is incorporated into the viral genome. Incorporation into the viral genome is thought to lead to chromosomal degradation and a reduced viral load for the host. To date, ITPase has not been demonstrated to have a direct role in ribavirin metabolism (although evidence suggests it may [50]), however it is thought to modulate overall nucleotide pools and *ITPA* status can affect treatment outcomes as discussed below [52].

#### ITPA variation affects outcome of thiopurine treatment

Several groups have shown that *ITPA* variation correlates with increased toxicity to thiopurines [53, 54, 56, 59–61]. Thiopurine therapy is widespread. Thiopurines, such as azathioprine or 6-mercaptopurine, are immunosuppressants that are commonly administered to organ transplant recipients [62], and patients undergoing treatment for inflammatory bowel disease [63], ulcerative colitis [64], Crohn’s Disease [65] and a variety of cancers [66–68]. For patients harboring *ITPA* variation, common side effects included rash, flu-like symptoms, pancreatitis, life threatening myelosuppression, and hepatotoxicity. Toxicity presumably occurs due to an increase of thiol-containing NTPs. Because these side effects can be quite devastating to the patient, dosage alteration or discontinuation of treatment is common for individuals with *ITPA* polymorphism [53, 59] (see references [36, 56, 57] for review). As a result, researchers have suggested including pre-therapeutic screening of patients for *ITPA* polymorphism to minimize the occurrence of drug toxicity [69–71]. Importantly, not all *ITPA* polymorphism is associated with thiopurine toxicity. For example, one group found that heterozygosity for the c.94C > A (p.Pro32Thr) allele was associated with adverse drug reactions, but both hetero- and homozygosity for the c.124 + 21A > C allele was not [59].

An alternative approach to alleviating thiopurine toxicity is to prescribed 6-thioguanine in place of azathioprine or 6-mercaptopurine. Bioactivation of 6-thioguanine proceeds via a thio-GMP intermediate (6-TGMP), rather than a thio-IMP intermediate (6-TIMP), thus by-passing the ITPase reaction (see Fig. 5a) [59]. Substitution with 6-thioguanine has been used successfully in a cohort of Crohn’s disease patients who demonstrated resistant to 6-mercaptopurine therapy [72].

One caveat to the involvement of ITPase in thiopurine toxicity is that some studies have not found a correlation between *ITPA* variation and thiopurine toxicity [73–76]. The conflicting data underscores the fact that many different gene products have a role in thiopurine metabolism (at least 20), and phenotypic variation among patients may mask effects of *ITPA* variation in certain studies [70]. Indeed, studies that do not find a correlation between *ITPA* variation and drug toxicity identify small sample size, ethnicity, drug interactions and the retrospective nature of their studies as shortcomings [73, 74, 76, 77]. In 2014, Matimba et al. used a “three-tiered” experimental strategy to overcome some of the shortcomings associated with clinical studies. This allowed them to positively identify and functionally validate an association between *ITPA* variation and an altered thiopurine response phenotype [70].

#### ITPA variation affects outcome of hepatitis C treatment

In 2010 and following studies, the c.94C > A (p.Pro32Thr) variant was shown to delay the development of anemia in patients treated for chronic hepatitis [52, 78, 79]. For the 170 million people affected with hepatitis C worldwide, one of the potential side effects for the standard treatment of pegylated interferon alpha and ribavirin is hemolytic anemia [80]. In a follow up study, researchers found that individuals harboring either the c.94C > A (p.Pro32Thr) or c.124 + 21A > C variations not only had delayed development of anemia, but also had a decreased need for ribavin dose reduction [51]. Recently, methods have been developed to use ITPase activity as an accurate predictor for the development of anemia during ribavirin treatment [81]. Based on these findings, it has been suggested that status of the *ITPA* locus, or ITPase activity, should be considered for patients beginning hepatitis C therapies which include ribavirin [51, 81–83]. In fact, an *ITPA* SNP-based index has been developed to advise healthcare workers in maximizing the tolerability of treatment regimen for patients with chronic hepatitis C [84].

While the mechanism for the protective effect of *ITPA* polymorphism is under debate, it seems likely that modulation of nucleotide pools plays a role. Ribavirin-induced anemia is thought to result from decreased erythrocyte ATP levels, which in turn affects ATP-dependent oxidative metabolism [85] and reduces the levels of GTP in the cell [25]. However, it has been speculated that for individuals with reduced ITPase activity, a rise in intracellular ITP concentrations might allow ITP to replace GTP as a cosubstrate for the adenylosuccinate synthetase reaction, thus allowing ATP biosynthesis to continue [25]. The rise in ATP levels subsequently allows GTP biosynthesis to continue. Therefore, an increased ITP concentration may allow erythrocyte nucleotide pools to maintain a more normal level which lowers the incidence of hemolytic anemia [86]. One caveat to this hypothesis is that adenylosuccinate synthetase activity has not been demonstrated in erythrocytes [81].

The mode of action of ribavirin requires bioactivation to the nucleotide ribavirin 5′-triphosphate (RBV-TP), which is subsequently incorporated in the viral genome and interferes with replication [52, 58]. Individuals with variant *ITPA* phenotypes have increased levels of RBV-TP in their red blood cells [50]. Importantly, the level of RBV-TP in cells corresponded to *ITPA* status, i.e. genotypes predicted to have lower ITPase activity had greater levels of RBV-TP [50]. Ribavirin has an electronegative oxygen (carbonyl) in approximately the same location as the 6-position carbonyl in inosine [31] (see Fig. 4). Taken together, it is likely that RBV-TP is a substrate for ITPase, but this has not been directly demonstrated in the current literature.

Overall, it appears that *ITPA* variation would alter the monophosphate to triphosphate ratio of ribavirin containing nucleotides. Consequently, it is possible that the mechanism for the observed delay in onset of anemia for patients with *ITPA* variation may involve a reduction in the level of the monophosphate form, or an increase in the level of the triphosphate form of ribavirin. Furthermore, the role of nucleoside diphosphate kinase should be considered. This enzyme is active in erythrocytes [87] and its reversible and promiscuous ping-pong mechanism essentially allows any NTP to provide the phosphorylation power to quickly convert any NDP into its corresponding NTP [25]. Therefore, in the case of *ITPA* variation, the rise in ITP and RBV-TP levels is expected to contribute to the pool of ATP equivalents in the cell.

*ITPA* variation has also been associated with reduced relapse risk and achieving sustained virological response (SVR) for hepatitis C patients [49]. Rembeck et al. found that for patients receiving a conventional 800 mg daily ribavirin dose and peginterferon-2α, those that had an *ITPA* status which was predicted to result in ITPase activity that was 60 % or less than wild-type, both relapse rate and SVR were favorably impacted. Importantly, the researchers observed no statistical difference in hemoglobin levels among patients achieving or not achieving SVR, suggesting that protection against onset of anemia is not related to an improved rate of SVR. For patients harboring *ITPA* variation, the authors suggested that imbalances in the NTP pools such as increased levels of ITP, along with decreased levels of GTP (due to inhibition of inosine monophosphate dehydrogenase by ribavirin) may result in increased incorporation of ITP into the viral genome. In turn, the increased level of inosine in viral RNA may trigger reduction of the viral load via an innate immune response [49].

#### ITPA mutation causes infantile encephalopathy

Kevelam et al. recently identified recessive *ITPA* mutation as a cause of early infantile encephalopathy [32]. The researchers used magnetic resonance imaging (MRI) and whole exome sequencing (WES) to link very rare, recessive mutation of the *ITPA* gene to a unique MRI pattern. Seven patients were identified who presented severe progressive microcephaly, seizures and developmental delays shortly after birth. Sadly, six of the seven patients died before 2.5 years of age. Cardiac abnormalities and severely reduced erythrocyte and fibroblast ITPase activity was observed, but purine and pyrimidine urine profiles were normal [32]. These phenotypes are consistent with high levels of brain and cardiac *ITPA* expression [5]. The homozygous genetic variation observed included gene deletions, as well as nonsense, frameshift and missense mutations [32]. All but the latter was predicted to significantly alter the primary structure of the resulting protein. In the case of the missense mutation, a nonfunctional protein with a substitution of Arg-178 with cysteine was predicted. An R178C substitution would alter a key residue in the substrate specificity pocket and this substitution is predicted to affect the ability of this residue to hydrogen bond with the 6-position keto oxygen of ITP (see above and Fig. 3) and would most likely reduce affinity for ITP [32]. Alternatively, because this point mutation introduces an additional cysteine residue into the primary protein structure, it is possible that improper disulfide bonds could form with one of the seven other cysteine residues during protein maturation. Perusal of x-ray crystallographic data [4] suggests that native ITPase quaternary structure does not contain any disulfide bonds, however, position 178 is in close proximity to Cys-116 in a neighboring beta strand (personal observation). Preliminary work with the R178C mutant ITPase indicates this protein has a low level of solubility when overexpressed in *E. coli* cells (unpublished data), however the occurrence of disulfide bond formation is not known. Because ITPase has been shown to be activated with DTT [5, 28], it is possible cysteine redox chemistry has a role in the enzyme structure and/or chemistry.

#### ITPA variation affects onset of tuberculosis

Recently, the *ITPA* gene has been identified as a susceptibility gene for young-onset tuberculosis (TB) [44]. Here, the researchers used next-generation sequencing (NGS) to identify single nucleotide polymorphisms in young TB patients from multi-case families and found two *ITPA* polymorphisms (g.19176G > A and c.94C > A (p.Pro32Thr)) to be associated with young TB patients. The g.19176G > A variant showed the strongest association. This variant is located in the 3′-UTR of the *ITPA* gene and it is hypothesized to modulate *ITPA* expression at the posttranscriptional level. *In silico* eQTL analysis using lymphoblastoid expression profile data indicates an increased level of expression for the minor 'A' allele form of this variant. Subsequently, it was hypothesized that a lower level of expression for the major 'G' allele may render individuals harboring this allele more susceptible to TB infection. Similarly, c.94C > A (p.Pro32Thr) polymorphism, which would cause reduced ITPase activity (see above), is also linked to increased susceptibility. Considering that the thymus is among the human tissues with highest *ITPA* expression [5], these results suggest that ITPase may have a role in the host immune system upon onset and progression of TB [44]. Therefore, this study introduces the idea that overexpression of the *ITPA* gene may have a protective effect.

#### A disease spectrum for ITPA variation

Vanderheiden first identified patients with an abnormal accumulation of erythrocyte ITP in the 1960s and suggested that these patients were ITPase deficient [88, 89]. In 2002 a report indicated that erythrocytes from c.94C > A (p.Pro32Thr) homozygous individuals showed 0 % ITPase activity, and, it was noted that there appeared to be no biological consequence of this absence [40]. Today it is clear that ITPase has an essential role in purine metabolism [32]. This is underscored by the fact that ITPase is so highly conserved among all branches of biology [12], and expressed in all mammalian tissues assayed [5]. The role of the protein in specific tissues appears to differ and measurement of activity in one specific tissue does not necessarily reflect activity of the whole organism. For instance, one study determined that low erythrocyte ITPase activity did correlate with lower activity in white blood cells and platelets, but the overall level of activity was considerably higher for these cells [90]. Over time, more and more reports revealed significant relationships between *ITPA* variation and altered drug regimen outcomes [52, 59–61, 78, 79]. Today, with two examples of lethality due to severe homozygous *ITPA* defects (*Itpa* knockout mouse data [6] and infantile encephalopathy data [32]), along with an example of increased fitness due to potential *ITPA* overexpression (TB susceptibility data [44]), it seems more likely that there is a spectrum of *ITPA*-related disease as Kevelam et al suggested [32]. Hence, most *ITPA* variation results in modulated *ITPA* expression/activity, rather than absence of ITPase, therefore, use of term “ITPase deficiency” in the literature to describe the majority of *ITPA* polymorphism appears to be a historical inaccuracy.

### Future directions

Considering that the current data indicates the phenotypic spectrum of *ITPA* defects ranges from mild drug toxicity to infantile death, it seems likely that therapies will emerge to modulate ITPase activity. Advances in DNA sequencing techniques, such as WES and NGS, and reduced cost of sequencing is likely to identify an increased number of instances where *ITPA* status contributes to neurological and cardiac disease as well as complications associated with inflammation, the immune system or other unknown *ITPA*-related diseases. Given the widespread use of thiopurines, and magnitude of individuals infected with hepatitis C, while considering that roughly one third of the world population has *ITPA* polymorphism, it seems plausible that development of these therapies will be met with considerable need.

Above, I have outlined examples where *ITPA* polymorphism/mutation is detrimental to patient outcomes (thiopurine toxicity/infantile death) and examples indicating that *ITPA* polymorphism is beneficial to patient outcomes (reduced anemia in hepatitis C patients, young-onset TB). Current treatment for the pharmacological related conditions includes dose modification, treatment of the side effects or discontinuation of the drug [54, 91]. Below, I will outline strategies which have potential to prevent the occurrence of negative outcomes for these patients. Therefore, ITPase serves as an interesting drug target because there is a need for both positive and negative modulation.

#### Positive modulation of ITPase activity

With the development of improved gene delivery systems, and gene editing tools one can envision a day when a bioengineered ITPase (with increased activity and improved substrate discrimination) or gene editing system (to enhance/correct ITPase function), can be delivered to a patient suffering from cardiac or neurological maladies due to very rare and severe mutation, or even possibly patients suffering from thiopurine toxicity due to common polymorphism. In the cell, this scenario would exacerbate existing ITPase activity and cause a much greater increase in specificity for (d)ITP or 6-TITP over the canonical (d)NTPs, thus reducing time spent with the unproductive binding and catalysis of adenine and guanine containing nucleotides. In *E.coli* cells, ITP levels are about 200 μM, whereas ATP and GTP are approximately 50- and 25-fold greater, respectively [92]. *K*_M_ values for ITPase with these substrates follow the same trend [2, 12]. Therefore, alteration of kinetic constants via protein engineering should result in improved ability of ITPase to discriminate between canonical and noncanonical purine containing nucleotides. This is expected to greatly enhance the ability of the enzyme to clear cells of excess (d)ITP or drug metabolite.

Towards this goal, we have identified three gain-of-function mutants [31]. Most notably, the E22A mutant shows a nearly two-fold enhancement in ITP hydrolyzing activity compared to wild-type, with a two- and five-fold decrease in hydrolyzing activity with GTP and ATP, respectively, compared to wild-type. Overall, we observe a roughly 10-fold enhancement over wild-type for discrimination between ITP and ATP. X-ray crystallography data suggests Glu-22 does not directly interact with the incoming nucleotide [4], but rather has an organizing role in the specificity pocket such as orienting the essential Arg-178 as discussed above (Fig. 3). Unfortunately, this protein has reduced stability in vivo and cannot complement ITPase defects in *E. coli*. Similarly, the K172A mutant exhibits a two-fold increase in ITP hydrolyzing activity and a greater than two-fold decrease in ATP or GTP hydrolyzing activity. In vivo, this mutant is more stable than the E22A mutant and it does have an ability to partially complement ITPase defects in *E. coli* [31]. Detailed biochemical and modeling data with these mutants will provide a platform for protein engineering experiments to continue improving ITPase activity and/or substrate discrimination with the aim of developing therapies to reverse thiopurine toxicity, neurological disorders, TB susceptibility and emerging *ITPA*-related disorders in patients affected by *ITPA* polymorphism.

In the case of the c.94C > A (p.Pro32Thr) ITPase, a portion of the reduction in enzyme activity has been attributed to reduced protein stability and rate of catalysis [33–35] (see above). This substitution affects an exterior loop of the protein, and substitution with an amino acid that does not have a tertiary peptide amine, renders the loop more flexible. Therefore, it has been speculated that a small molecule could be designed which would bind to this region of the protein and help stabilize this exterior loop [7].

#### Negative modulation of ITPase activity

Because reduced ITPase activity results in improved outcomes for patients undergoing ribavirin treatment [52], it is plausible that competitive ITPase inhibitors could be developed to help reduce anemia in individuals not affected by *ITPA* polymorphism. Therefore, molecules which inhibit ITPase activity are expected to have clinical significance. Considering that that the substrate specificity pocket has exquisite complementarity to the nucleobase of ITP and strongly favors electronegativity at the 6-position [4], competitive inhibitor molecule design should focus on nucleotides or nucleobases where the nucleobase has chemical properties similar to hypoxanthine. Alternatively, irreversible inhibitors could be designed to have structures that covalently attach to residues lining this pocket, or have such low dissociation constants that they irreversibly bind to this pocket. Because of the unique role ITPase has in the cell, the landscape of the substrate specificity site is expected to be unique, which would reduce the likelihood of cross-reactivity with ATP- and GTPases. Noncleavable substrates, such as inosine containing phosphoramidites, may prove advantageous as well, but would have greater difficulty entering the cell [25].

One confounding fact of ITPase biochemistry is that substrate inhibition has been demonstrated for two mammalian homologs with elevated concentrations of substrate [2, 5, 28]. Although substrate inhibition was first demonstrated over 45 years ago [28], the significance of this mechanism remains unknown. It seems likely this effect may be due to specific biochemical roles for ITPase in purine metabolism in certain tissues at certain times. Currently there is at least one example which indicates that ITPase is not subject to substrate inhibition in vivo [37], suggesting that a cellular component can reverse this phenomenon. To date this issue remains completely unresolved in the literature. Regardless, the effect of substrate inhibition with purified/recombinant ITPase is strong and renders mammalian ITPase nearly inactive when ITP concentrations are in the high micromolar range [2]. In fact, substrate inhibition occurs at elevated levels of ATP (unpublished observations) and GTP [28] as well. Overall, the possibility of negative allosteric binding sites should be considered when designing small molecule inhibitors for ITPase. Design of competitive and noncompetitive inhibitory molecules awaits more rigorous biochemical characterization and molecular modelling of ITPase.

## Conclusions

Recent studies demonstrate disparate examples of disease related to *ITPA* polymorphism/mutation. Recent biochemical studies have begun to elucidate the mechanism ITPase uses to discriminate between canonical and noncanonical purine containing NTPs, and determine the underlying mechanism of disease state. In general, ITPase is an understudied protein and a knowledge gap exists in the basic science of ITPase biochemistry. Currently, seven *ITPA* variants/mutants are known to affect clinical outcomes. *ITPA* status can have beneficial or detrimental consequences, depending on the clinical setting. Drug toxicity occurs in patients with *ITPA* variation undergoing thiopurine therapy, whereas development of anemia in the treatment of hepatitis C is delayed for individuals with *ITPA* polymorphism. The current understanding is that these individuals retain a functional enzyme, however, the enzyme expression is reduced (c.124 + 21A > C), and in some cases catalysis and stability is reduced as well (c.94C > A (p.Pro32Thr)). To date, several preliminary reports have indicated a need to determine *ITPA* status, or enzyme activity, prior to treatment for conditions where *ITPA* status has been shown to affect patient outcomes, however, no large scale prospective cohort studies have been performed to validate these results. ITPase enzyme activity is directly related to *ITPA* status, and individuals harboring homozygous *ITPA* variation experience the greatest effects. Instances of mutation, where the protein length (c.264-607_295 + 1267del1906, c.359_366dupTCAGCACC, c.452G > A (p.Trp151Stop)) and/or functionality (c.532C > T (p.Arg178Cys)) is grossly altered are very rare and homozygosity results in infantile encephalopathy and lethality. On the other hand, *ITPA* variation which is predicted to increase protein expression is thought to reduce susceptibility to tuberculosis. Consideration of these examples suggests that there is a spectrum of *ITPA*-related disease. Future studies concerning both positive and negative modulation of ITPase activity will meet the needs of patients suffering from *ITPA*-related disease.

## Abbreviations

(d)ITP: (deoxy)inosine 5′-triphosphate
(d)NMP: (deoxy)nucleoside 5′-monophosphate
(d)NTP: (deoxy)nucleoside 5′-triphosphate
3′-UTR: 3′-untranslated region
6-TITP: 6-thio-inosine 5′-triphosphate
AMP: Adenosine 5′-monophosphate
AMPPNP: Adenylylimidodiphosphate
DTT: Dithiothreitol
GMP: Guanosine 5′-monophosphate
IMP: Inosine 5′-monophosphate
ITPase: Inosine 5′-triphosphate pyrophosphatase
MRI: Magnetic resonance imaging
NGS: Next-generation sequencing
PP_i_: Pyrophosphate
RBV-TP: Ribavirin 5′-triphosphate
SNP: Single nucleotide polymorphism
TB: Tuberculosis
WES: Whole exome sequencing
